# Integrating the GRACE Score with the Ceramide Risk Score Enhances the Predictive Accuracy of Major Adverse Cardiac Events in Patients with Acute Coronary Syndrome Undergoing Percutaneous Coronary Intervention

**DOI:** 10.31083/RCM25984

**Published:** 2024-12-31

**Authors:** Xiaofei Wang, Chengzhe Liu, Fu Yu, Zizhuo Zhang, Jiale Wang, Xiaoyu Shi, Tianyou Xu, Qiang Deng, Liping Zhou, Wanyue Sang, Hong Jiang, Lilei Yu

**Affiliations:** ^1^Department of Cardiology, Renmin Hospital of Wuhan University, 430060 Wuhan, Hubei, China; ^2^Hubei Key Laboratory of Autonomic Nervous System Modulation, 430060 Wuhan, Hubei, China; ^3^Cardiac Autonomic Nervous System Research Center of Wuhan University, 430060 Wuhan, Hubei, China; ^4^Institute of Molecular Medicine, Renmin Hospital of Wuhan University, 430060 Wuhan, Hubei, China; ^5^Hubei Key Laboratory of Cardiology, 430060 Wuhan, Hubei, China; ^6^Taikang Center for Life and Medical Sciences, Wuhan University, 430060 Wuhan, Hubei, China; ^7^Cardiovascular Research Institute, Wuhan University, 430060 Wuhan, Hubei, China

**Keywords:** acute coronary syndrome, GRACE score, ceramide, major adverse cardiac events, quantitative flow ratio

## Abstract

**Background:**

Ceramide, a key molecule in sphingolipid metabolism, is recognized as a standalone predictor of long-term major adverse cardiac events (MACE). We explore if integrating the global registry of acute coronary events (GRACE) score with the ceramide risk score (ceramide test 1, CERT1) improves MACE prediction in patients with acute coronary syndrome (ACS) undergoing percutaneous coronary intervention (PCI).

**Methods:**

This cohort study included 210 participants with ACS undergoing PCI. MACE was defined as the recurrence of non-fatal acute myocardial infarction, repeat coronary revascularization procedures (PCI or coronary artery bypass grafting, CABG), or death excluding the initial event qualifying the patient for the study. The cumulative incidence of MACE was analyzed using the Kaplan-Meier method. Both univariate and multivariate Cox regression analyses identified MACE predictors. The predictive accuracy of combining the GRACE score with the CERT1 score was assessed using the area under the receiver operating characteristic curve (AUC), integrated discrimination improvement (IDI), and net reclassification improvement (NRI).

**Results:**

During the 12-month follow-up period, 35 of the 210 participants experienced a MACE. The Kaplan-Meier analysis revealed a significant variation in MACE incidence stratified by the CERT1 score (χ^2^ = 21.344, *p* < 0.001). Multivariate Cox regression analysis identified low-density lipoprotein (*p* = 0.002), quantitative flow ratio (*p* = 0.013), the CERT1 score (*p* = 0.005), and the GRACE score (*p* = 0.007) as independent predictors for MACE. Integrating the GRACE score with the CERT1 score improved prediction accuracy, raising the AUC from 0.733 to 0.834. This adjustment provided a more precise risk reclassification and discrimination between patients likely and unlikely to experience MACE (NRI: 0.526, *p* = 0.004; IDI: 0.120, *p* < 0.001).

**Conclusions:**

The CERT1 score independently predicts long-term MACE for individuals diagnosed with ACS undergoing PCI. Including the CERT1 score significantly enhances the GRACE score's capacity to risk-stratify these patients.

**Clinical Trial Registration:**

Registration number: ChiCTR2300068491 (https://www.chictr.org.cn/showproj.html?proj=180370).

## 1. Introduction

Cardiovascular diseases are an important global public health problem with incidences rising annually [1]. Acute coronary syndrome (ACS), one of the most severe subtypes, presents a wide range of prognoses depending on the various pathophysiological elements. Early risk stratification is crucial for determining appropriate treatment strategies, which can prevent overtreatment, reduce healthcare costs, and improve patient outcomes [2, 3, 4]. The global registry of acute coronary events (GRACE) score is a widely utilized tool for predicting short- and long-term outcomes in ACS patients, including major adverse cardiovascular events (MACE) [5, 6]. While sphingolipid metabolism has been shown to play a critical role in ACS progression and prognosis [7], the GRACE score does not account for biological markers related to this pathway, limiting its predictive accuracy.

Ceramide plays a pivotal role in sphingolipid metabolism, particularly in the context of cardiovascular disease. It tends to accumulate in areas where low-density lipoprotein (LDL) deposits *in vivo*, facilitating the penetration of lipoproteins through the vascular wall, contributing to the destabilization of atherosclerotic plaques [8, 9]. Recent studies have demonstrated the critical role of ceramide in predicting MACE in individuals with ACS [10, 11]. However, the potential application of ceramide as a key biomarker to enhance the predictive accuracy of the GRACE score, specifically for long-term outcomes in ACS patients undergoing percutaneous coronary intervention (PCI), is still under investigation.

In this study, we combined the ceramide and GRACE risk scores into a novel metric to test its capacity to predict the occurrence of MACE in patients with ACS undergoing PCI. Furthermore, we assessed whether this integration enhances the prognosis assessment following patient discharge.

## 2. Materials and Methods

### 2.1 Study Design and Participants

**Fig. 1.**
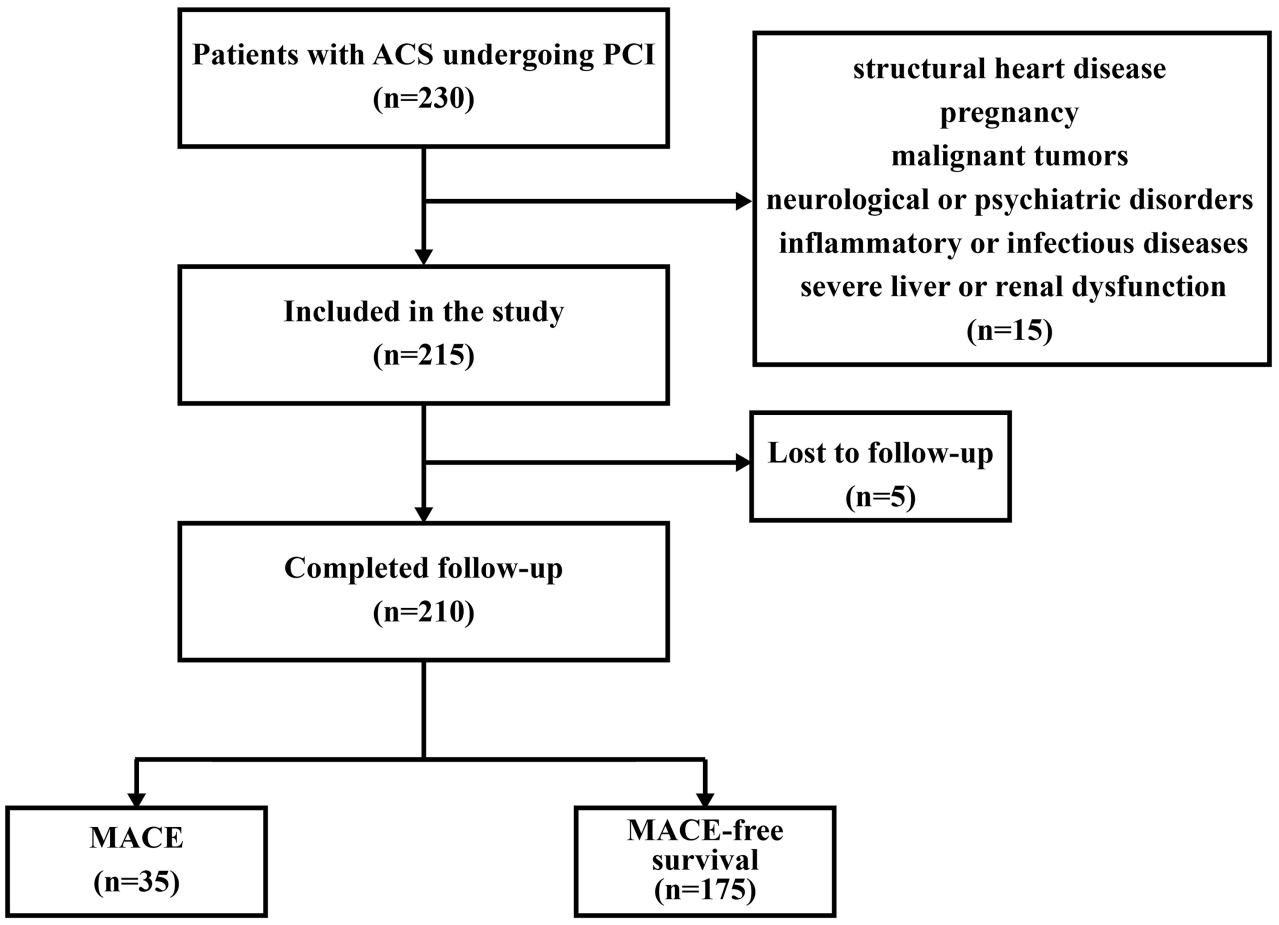
**Patient enrollment, inclusion, and exclusion criteria**. ACS, acute coronary syndrome; PCI, percutaneous coronary intervention; MACE, major adverse cardiovascular events.

We carried out a prospective observational cohort study at the Renmin Hospital of Wuhan University (Wuhan, China), enrolling 210 participants with ACS undergoing PCI between June 2022 and June 2023. The primary eligibility criteria for the study included patients scheduled to undergo PCI with a confirmed diagnosis of ACS, which covered the full spectrum of conditions including unstable angina (UA), non-ST-segment elevation myocardial infarction (NSTEMI), and ST-segment elevation myocardial infarction (STEMI). The diagnosis and treatment of ACS were determined in accordance with the latest guidelines established by the European Society of Cardiology [12]. The exclusion criteria included participants with structural heart disease, pregnancy, malignant tumors, neurological or psychiatric disorders, inflammatory or infectious diseases, and severe liver or renal dysfunction. A flowchart of the participant recruitment process can be found in Fig. 1. The research received approval from the Ethics Committee of Renmin Hospital of Wuhan University, and participating patients gave informed consent.

### 2.2 Laboratory Examinations

Peripheral venous blood samples were collected from fasting participants the morning after the PCI procedure. Routine indicators tested included blood components, plasma lipids, liver function, and kidney function.

### 2.3 Determination and Calculation of Ceramide

Fasting venous blood was collected from each participant, and the whole blood was centrifuged two hours after collection (1600 g, 10 minutes) to separate the serum. High-performance liquid chromatography in tandem with mass spectrometry (ACQUITY UPLC I-CLASS PLUS System (Waters, Milford, MA, USA) and AB Sciex QTRAP 4500 (SCIEX, Framingham, MA, USA)) was used to determine the content of four types of ceramides (Cer [d18:1/16:0], Cer [d18:1/18:0], Cer [d18:1/24:1], and Cer [d18:1/24:0]) in the serum.

The ceramide risk score, known as the CERT1 score, incorporates specific types of ceramides—Cer (d18:1/16:0), Cer (d18:1/18:0), and Cer (d18:1/24:1)—and their ratio to Cer (d18:1/24:0). These ceramides and ratios are used as variables to determine the overall score. The CERT1 score is quantified on a scale ranging from 0 to 12, which is divided into quartiles. Points are assigned based on the concentration of these ceramides or their ratios: the top 25% (highest risk quartile) receive 2 points, the next 25% receive 1 point, and the lowest 50% (lowest risk half) receive no points [10]. Based on the total points scored, patients are categorized into low-risk (scores 0–2) or intermediate to high-risk (scores 3–12).

### 2.4 Calculation of the GRACE Score

The determination of the GRACE score is based on a variety of clinical parameters, including age, presence of heart failure, heart rate, ST-segment depression, blood pressure, kidney function as indicated by creatinine levels, in-hospital procedures like PCI and CABG, past episodes of myocardial infarction, and elevated cardiac biomarkers [13, 14]. For an accurate calculation of one’s GRACE score, a web-based tool is available at the University of Massachusetts Medical School’s website: https://www.outcomes-umassmed.org/risk_models_grace_orig.aspx.

### 2.5 Definition of MACE and Follow-up

MACE is defined as the recurrence of non-fatal acute myocardial infarction, repeat procedures for coronary revascularization (PCI or CABG), or death excluding the initial event that qualified the patient for the study. Follow-up of the enrolled patients was conducted via telephone interviews or by reviewing electronic health records. The study’s observation phase concluded upon the first instance of a MACE. Data collection and monitoring continued up to May 30, 2024.

### 2.6 Quantitative Flow Ratio Computation

Patients with ACS underwent post-PCI quantitative flow ratio (QFR) measurements conducted by two interventional cardiologists, unaware of the clinical details, utilizing the AngioPlus platform (Pulse Medical Imaging Technology, Shanghai, China). Standard coronary angiography (CAG) images were collected. Two CAG images with an angle difference greater than 25° were transferred via the imaging data transfer system to the AngioPlus system. The QFR values are depicted on a pullback curve for each of the three major vessels.

### 2.7 Statistical Analysis

Continuous variables were obtained from the average value accompanied by the standard deviation (SD) or by median values alongside their interquartile ranges (IQR). Categorical variables were presented in terms of counts and percentage distributions. Comparisons between two groups were made using independent samples *t*-tests or Mann-Whitney U tests. Comparison of categorical data was performed using the chi-square test (χ^2^ test). The cumulative MACE curves were assessed through the application of the Kaplan-Meier method. Univariate and multivariate Cox regression analyses were employed to elucidate the independent risk factors associated with MACE among individuals with ACS. The discriminatory power of various predictive models regarding clinical endpoints was assessed by examining the area under the receiver operating characteristic (ROC) curve, integrated discrimination improvement (IDI) and net reclassification improvement (NRI). Comprehensive statistical evaluations were conducted using the SPSS software, version 26.0 (IBM Corp, Armonk, NY, USA). A *p*-value < 0.05 was regarded as indicative of statistical significance.

## 3. Results

### 3.1 Baseline Patient Characteristics

In total, the study enrolled 210 patients who were stratified into two risk levels. Participants with CERT1 scores of 0 to 2 points were characterized as low risk, while those with 3 to 12 points were categorized as intermediate to high risk. Our results showed that high levels of body mass index (BMI, *p* < 0.001), high-sensitivity C-reactive protein (Hs-CRP, *p* < 0.001), hemoglobin A1c (HbA1C, *p* < 0.001), and LDL (*p* < 0.001) were more common in people with moderate to high risk. In addition, such patients showed a higher GRACE score (*p* = 0.015), and a significantly increased rate of MACE (*p* < 0.001). This group also had a reduced estimated glomerular filtration rate (eGFR, *p* = 0.002), and a lower QFR post-PCI (*p* < 0.001). The baseline characteristics of the participants are shown in Table 1.

**Table 1.** **Characteristics of 210 participants with ACS undergoing PCI categorized by CERT1 score**.

Variable		Low risk group	Intermediate to high- risk group	*p* value
		(CERT1: 0–2 points)	(CERT1: 3–12 points)	
		(n = 92)	(n = 118)	
Baseline characteristic				
	Age, years	61.06 (55.44, 64.96)	61.88 (57.83, 66.38)	0.367
	Sex, male	68 (73.91%)	89 (75.42%)	0.803
	BMI, kg/m^2^	24.67 ± 2.87	27.40 ± 2.03	<0.001
	SBP, mm Hg	125.28 ± 16.14	128.07 ± 15.63	0.207
	Heart rate, bpm	70.34 (62.88, 78.87)	72.13 (65.00, 78.98)	0.230
	Hypertension	56 (60.87%)	73 (61.86%)	0.883
	Smoking	28 (30.43%)	37 (31.36%)	0.886
	Prior MI	13 (14.13%)	27 (22.88%)	0.109
	Prior PCI or CABG	18 (19.57%)	25 (21.19%)	0.773
	Family history of CAD	26 (28.26%)	36 (30.51%)	0.723
Biochemical indicators				
	Hs-CRP, mg/L	1.25 (0.48, 1.88)	2.01 (1.02, 2.85)	<0.001
	eGFR, mL/min/1.73 m^2^	102.11 ± 32.08	89.22 ± 26.43	0.002
	Pro-BNP, ng/mL	808.07 ± 131.54	836.26 ± 154.44	0.163
	PLT count, 10^9^/L	204.44 ± 61.45	198.58 ± 61.03	0.492
	WBC count, 10^9^/L	7.67 ± 3.08	7.96 ± 2.49	0.473
	Neutrophile count, 10^9^/L	4.03 (2.55, 5.47)	4.41 (2.27, 6.37)	0.451
	HbA1C (%)	5.93 ± 0.97	6.49 ± 1.12	<0.001
	LVEF (%)	58.35 (50.42, 63.59)	55.84 (47.75, 62.80)	0.111
	TC, mmol/L	4.01 ± 0.75	4.17 ± 0.83	0.150
	TG, mmol/L	1.46 ± 0.28	1.54 ± 0.37	0.062
	HDL, mmol/L	0.99 ± 0.29	0.97 ± 0.39	0.696
	LDL, mmol/L	2.33 ± 0.92	2.89 ± 0.91	<0.001
QFR		0.94 (0.90, 0.97)	0.86 (0.79, 0.90)	<0.001
GRACE score		81.00 (73.00, 100.00)	90.00 (79.00, 124.00)	0.015
MACE (%)		3 (3.26%)	32 (27.12%)	<0.001

Data were presented as mean ± SD or n (%). BMI, body mass index; SBP, systolic blood pressure; Prior MI, prior myocardial infarction; Prior PCI or CABG, prior percutaneous coronary intervention or coronary artery bypass grafting; Family history of CAD, family history of coronary artery disease; Hs-CRP, high-sensitivity C-reactive protein; eGFR, estimated glomerular filtration rate; Pro-BNP, pro-B-type natriuretic peptide; PLT, platelets; WBC, white blood cell; HbA1C, hemoglobin A1c; LVEF, fraction left ventricle ejection; TC, total cholesterol; TG, triglycerides; HDL, high-density lipoprotein cholesterol; LDL, low-density lipoprotein; QFR, quantitative flow ratio; GRACE score, global registry of acute coronary events score; MACE, major adverse cardiac events; ACS, acute coronary syndrome; CERT1, ceramide risk.

### 3.2 The Connection between the CERT1 Score and MACE

**Fig. 2.**
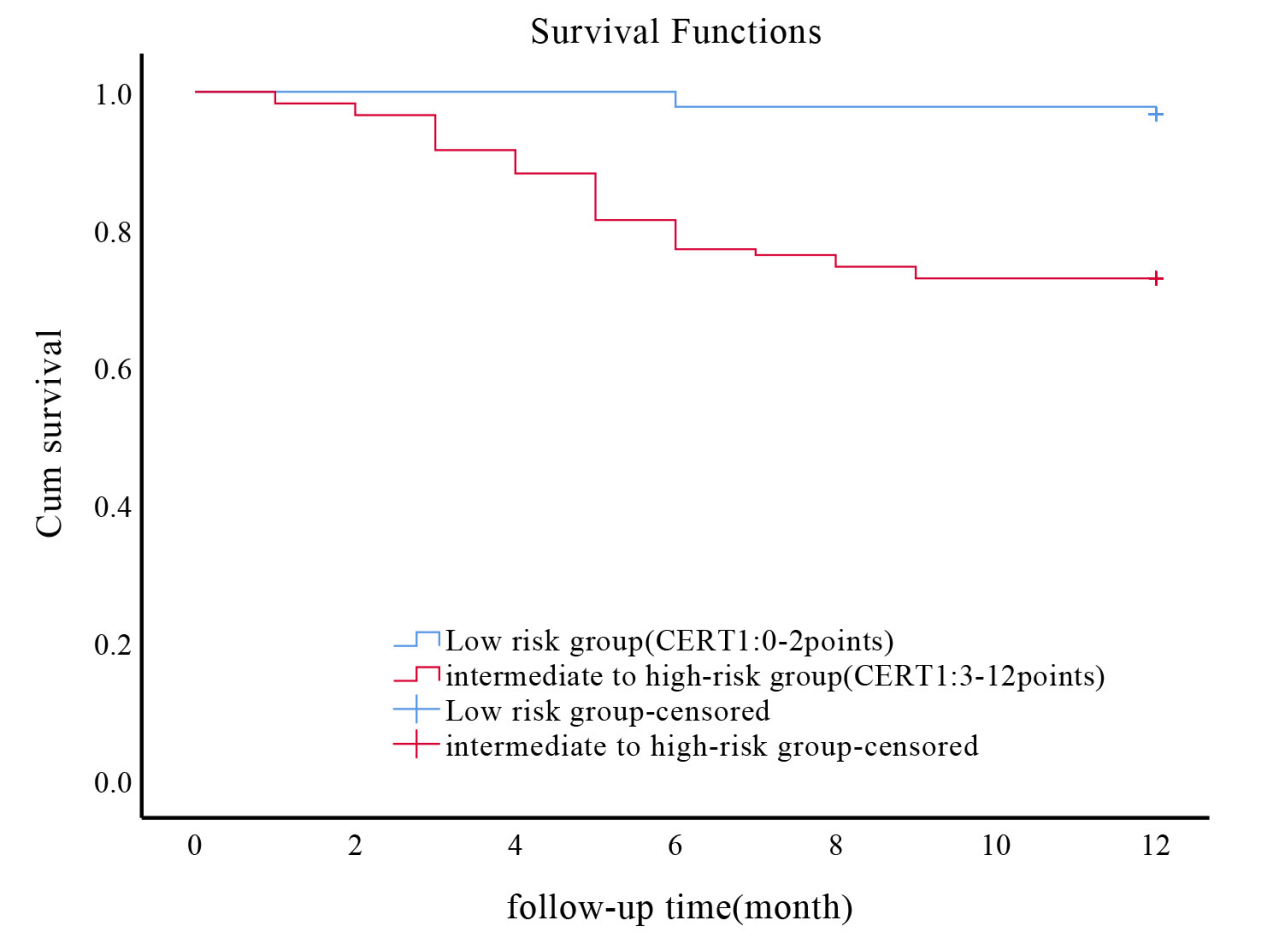
**Association between CERT1 score and incidence of MACE in ACS patients undergoing PCI**. Kaplan-Meier survival curves illustrating the incidence of MACE in ACS patients following PCI, stratified by CERT1 scores. CERT1, ceramide risk; MACE, major adverse cardiovascular events; ACS, acute coronary syndrome; PCI, percutaneous coronary intervention.

Of the 210 participants, MACE occurred in 35: 3 in the low-risk group (3.26%, n = 92) and 32 in the intermediate and high-risk group (27.12%, n = 118). Notably, seven days following surgery, two individuals in the intermediate to high-risk group experienced MACE, whereas no MACE events were observed in the low-risk group. This suggests that the CERT1 score could serve as an early MACE predictive risk factor for post-surgery risk in ACS patients undergoing PCI. Kaplan-Meier analysis revealed a significant difference in MACE cases among ACS patients receiving PCI stratified by their CERT1 scores (χ^2^ = 21.344, *p* < 0.001, as illustrated in Fig. 2). Higher CERT1 scores correlated with an increased probability of MACE.

### 3.3 Prognostic Indicators for MACE Occurrence

The univariate Cox regression analysis identified several potential predictive markers for MACE in ACS patients undergoing PCI. Hs-CRP was found to be significantly associated with MACE risk (*p *= 0.030), suggesting its role as an inflammatory biomarker in post-PCI outcomes. Additionally, LDL levels (*p *= 0.004) and QFR (*p* < 0.001) emerged as key contributors to MACE prediction, reflecting the importance of lipid management and coronary lesion severity. Notably, both the CERT1 score (*p* < 0.001) and the GRACE score (*p* < 0.001) were predictive, underscoring their combined value in risk stratification. These findings highlight the multifactorial nature of MACE risk in this patient population, as detailed in Table 2.

**Table 2.** **Univariate and multivariate COX regression analysis of MACE**.

Indicators	Univariate				Multivariate			
	*p*-value	HR	95% CI		*p*-value	HR	95% CI	
Age, years	0.087	1.039	0.995	1.085				
Sex, male	0.993	1.003	0.470	2.141				
BMI, kg/m^2^	0.073	1.122	0.990	1.271				
SBP, mm Hg	0.429	1.008	0.988	1.029				
Heart rate, bpm	0.417	1.011	0.985	1.038				
Hypertension	0.932	1.030	0.519	2.045				
Smoking	0.762	0.893	0.429	1.859				
Prior MI	0.799	0.892	0.370	2.149				
Prior PCI or CABG	0.891	0.943	0.412	2.160				
Family history of CAD	0.870	0.940	0.452	1.958				
Hs-CRP, mg/L	0.030	1.363	1.031	1.803	0.573	1.093	0.802	1.490
eGFR, mL/min/1.73 m^2^	0.917	0.999	0.988	1.011				
Pro-BNP, ng/mL	0.442	1.001	0.999	1.003				
PLT count, 10^9^/L	0.569	0.998	0.993	1.004				
WBC count, 10^9^/L	0.915	1.007	0.893	1.134				
Neutrophile count, 10^9^/L	0.112	1.097	0.979	1.231				
HbA1C (%)	0.052	1.354	0.997	1.840				
LVEF (%)	0.846	0.997	0.966	1.028				
TC, mmol/L	0.504	1.152	0.761	1.744				
TG, mmol/L	0.466	1.430	0.547	3.741				
HDL, mmol/L	0.598	0.771	0.294	2.027				
LDL, mmol/L	0.004	1.606	1.162	2.220	0.002	1.728	1.215	2.456
QFR	<0.001	0.000	0.000	0.003	0.013	0.008	0.000	0.361
CERT1 score	<0.001	1.334	1.207	1.474	0.005	1.188	1.052	1.341
GRACE score	<0.001	1.026	1.015	1.036	0.007	1.016	1.004	1.028

MACE, major adverse cardiac events; HR, hazard ratio; 95% CI, 95% confidence interval; BMI, body mass index; SBP, systolic blood pressure; Prior MI, prior myocardial infarction; Prior PCI or CABG, prior percutaneous coronary intervention or coronary artery bypass grafting; Family history of CAD, family history of coronary artery disease; Hs-CRP, high-sensitivity C-reactive protein; eGFR, estimated glomerular filtration rate; Pro-BNP, pro-B-type natriuretic peptide; PLT, platelets; WBC, white blood cell; HbA1C, hemoglobin A1c; LVEF, fraction left ventricle ejection; TC, total cholesterol; TG, triglycerides; HDL, high-density lipoprotein cholesterol; LDL, low-density lipoprotein cholesterol; QFR, quantitative flow ratio; GRACE score, global registry of acute coronary events score; CERT1 score, ceramide risk score. The corresponding related index group is *p* < 0.05.

The multivariate Cox regression model was used to assess Hs-CRP, LDL, and QFR levels as well as CERT1 and GRACE scores. After adjusting for multiple confounding elements, it was determined that LDL (*p* = 0.002) and QFR (*p* = 0.013) levels as well as scores for CERT1 (*p* = 0.005) and GRACE (*p* = 0.007) were crucial independent prognostic indicators for MACE.

### 3.4 The Capability of the CERT1 Score Alongside the GRACE Score in Predictions of MACE

**Fig. 3.**
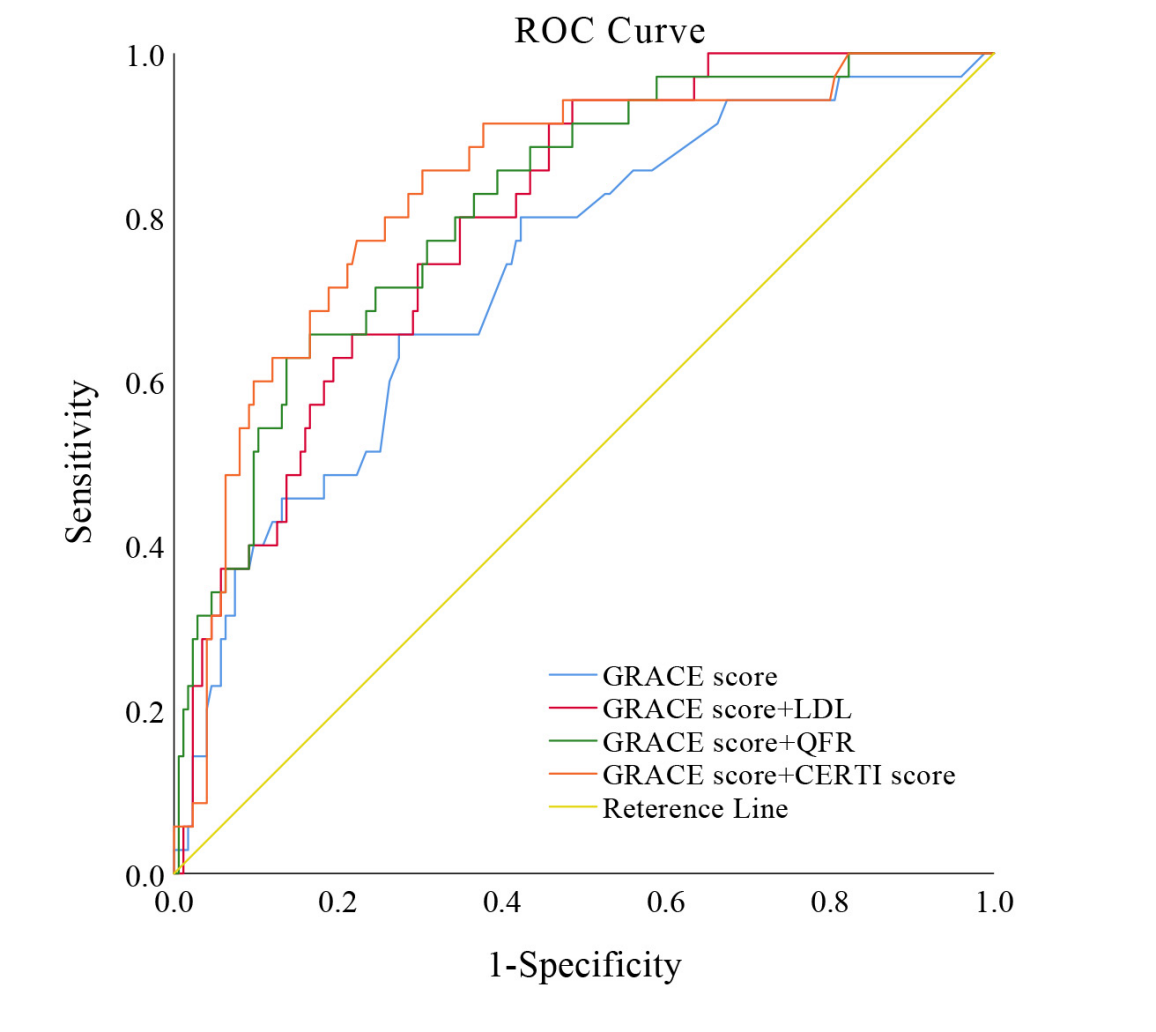
**Enhanced predictive accuracy of MACE models integrating CERT1 and GRACE scores**. This figure illustrates the enhanced discrimination and reclassification abilities of predictive models for MACE in ACS patients undergoing PCI. The model integrating CERT1 and GRACE scores shows superior predictive performance, confirming its value in clinical decision-making. ROC curve, receiver operating characteristic curve; GRACE score, global registry of acute coronary events score; LDL, low-density lipoprotein; QFR, quantitative flow ratio; CERT1 score, ceramide risk score; MACE, major adverse cardiac events.

In our study, LDL, QFR, and the CERT1 score each demonstrated a crucial, independent role in predicting the occurrence of MACE in patients with ACS undergoing PCI. Consequently, we evaluated each variable independently for its ability to predict long-term MACE risk, alongside the GRACE score. Integrating the CERT1 score with the GRACE score significantly enhanced the precision of MACE predictions, correlating with an increase in the area under the receiver operating characteristic curve (AUC) from 0.733 for the GRACE score alone to 0.834 when combined with the CERT1 score (Fig. 3).

The model’s efficacy surpassed the AUC of 0.798 attained when LDL was merged with the GRACE score, and it exceeded the AUC of 0.815 obtained when QFR was combined with the GRACE score. Moreover, the predictive effectiveness of various models was measured through NRI and IDI metrics. Integrating the CERT1 score into the GRACE score not only improved the predictive precision for subsequent MACE but also resulted in more effective outcomes in both net reclassification and combined discrimination. Specifically, the results showed an NRI of 0.526 (*p* = 0.004) and an IDI of 0.120 (*p* < 0.001), compared to conventional measures like LDL (NRI: 0.331, *p* = 0.068; IDI: 0.043, *p* = 0.062) and QFR (NRI: 0.503, *p* = 0.006; IDI: 0.103, *p* < 0.001; Table 3).

**Table 3.** **Comparative predictive effectiveness of various models for MACE incidence during follow-up**.

Model	GRACE score	GRACE score+LDL	GRACE score+QFR	GRACE score+CERTl score
AUC	0.733	0.798	0.815	0.834
*p*-value	<0.001	<0.001	<0.001	<0.001
95% CI	0.642–0.823	0.726–0.869	0.741–0.889	0.761–0.907
Sensitivity	65.71%	94.29%	65.71%	85.71%
Specificity	72.57%	51.43%	83.43%	69.71%
Youden index	0.383	0.457	0.491	0.554
NRI	-	0.331	0.503	0.526
*p*-value	-	0.068	0.006	0.004
IDI	-	0.043	0.103	0.120
*p*-value	-	0.062	<0.001	<0.001

MACE, major adverse cardiac events; GRACE score, global registry of acute coronary events score; LDL, low-density lipoprotein; QFR, quantitative flow ratio; CERT1 score, ceramide test 1; AUC, the area under the receiver operating characteristic curve; 95% CI, 95% confidence interval; NRI, net reclassification improvement; IDI, integrated discrimination improvement. The corresponding related index group is *p* < 0.05.

## 4. Discussion

Our results demonstrate that the combination of the CERT1 score and the GRACE score leads to a novel metric with the ability to independently predict clinical outcomes in patients with ACS undergoing PCI. This integrated approach enhances the predictive power of the GRACE score more effectively than traditional lipid markers (LDL) and imaging techniques (QFR). This improvement is expected to help clinicians identify personalized therapeutic strategies for their patients, thereby optimizing treatment outcomes.

Increasing evidence supports the notion that timely and comprehensive individual risk stratification for patients with ACS undergoing PCI can facilitate personalized treatments. This approach not only promises to improve outcomes for the high-risk patients but also aims to reduce the financial burden on those at low risk. According to current clinical practice guidelines, the use of the GRACE score is recommended for predicting short term and long-term prognostic outcomes of ACS patients undergoing PCI. However, when analyzed using the ROC curve, the AUC for the GRACE score alone stands at 0.733. This limited predictive power may be attributed to the omission of certain underlying risk factors in the existing model. Many researchers have sought to find new biomarkers to improve prognostic accuracy by adding C-reactive protein [15], HbA1C [16], TyG index [17], triglyceride-glucose index [18], pro-brain natriuretic peptide (pro-BNP) [19], and nutritional risk index [20] to the GRACE score. These efforts reflect the ongoing quest to refine risk assessment tools for better management of ACS patients.

A study has shown that hyperlipidemia is a major determinant of clinical prognosis in patients with ACS [21]. It is now widely accepted that strategies aimed at lowering lipid levels improve long-term cardiovascular outcomes and stabilize atherosclerotic plaques in ACS subjects [22, 23, 24, 25]. LDL is a standard indicator for blood lipids in clinical practice, and is a simple manifestation of lipid metabolism. This traditional lipid marker, however, is still not sufficient to completely represent the complexity of lipid metabolism. Findings from a large-scale cohort study indicated that higher CERT1 scores correlate with increased cardiovascular mortality rates among individuals with ACS, outperforming LDL levels as a prediction metric [10]. In our study, the addition of the CERT1 score to the GRACE score not only surpassed LDL in predictive ability but also significantly enhanced the capability to identify long-term significant cardiovascular complications in individuals with ACS following PCI. This underscores the value of incorporating the CERT1 score into existing prognostic frameworks to improve outcome predictions.

Both in-stent restenosis and residual stenosis may lead to MACE following PCI in patients with ACS. QFR is highly accurate in predicting whether coronary artery stenosis will lead to ischemia, and several studies have validated its ability to predict the risk of MACE [26, 27, 28]. Our study adds to this body of knowledge by demonstrating that the integrating the CERT1 score with the GRACE score not only provides a superior predictive ability for long-term MACE among ACS patients undergoing PCI, but also surpasses the predictive accuracy of the imaging biomarker QFR. This finding highlights the potential of combining biochemical and clinical risk scores to enhance prognostic assessments in this patient population.

Disrupted sphingolipid metabolism is closely related to the occurrence and development of ACS [29, 30]. Ceramide is a key component of sphingolipid metabolism [31, 32]. Ceramide stimulates the aggregation of LDL-C, facilitating the infiltration of lipoproteins into the vascular wall, increasing the transendothelial permeability for lipoproteins [33, 34, 35]. A key consequence is an increased risk of vascular occlusion reoccurring in patients with ACS that have had PCI. Under these circumstances, ceramide acts as a second messenger for cytokine induction, leading inflammatory immune cells to the plaque [31, 36]. This in turn can cause the plaque to rupture and ultimately produce an acute myocardial infarction along with related complications. Consequently, we propose that integrating the CERT1 score with the GRACE score can improve risk stratification and provide an important additional prognostic inform in patients with ACS undergoing PCI.

There are several important limitations of the study that must be considered. First, the research was conducted using a single-center, small-sample cohort, which may limit the generalizability of our results to more diverse patient populations. Future research should aim to incorporate multiple centers with heterogeneous patient groups for further validation. Second, our study only enrolled ACS patients undergoing PCI, thus the results may not be applicable to ACS patients undergoing alternative therapies or who are on medication. Third, measuring serum ceramide concentrations requires specialized laboratory equipment and technology, which can be costly and limit its clinical applicability. However, as diagnostic techniques improve and technologies such as mass spectrometry become more commonplace, it is expected that the costs associated with ceramide testing will decrease. Additionally, as our understanding of ceramide’s role in the pathophysiology of cardiovascular diseases deepens, routine testing for ceramide levels in clinical settings is anticipated to become more commonplace. Fourth, the addition of the CERT1 score to the GRACE score significantly enhanced prediction of MACE over the 1-year follow-up period. However, this integration slightly reduced the model’s specificity, potentially leading to an increase in false positive rates, potentially resulting in wrongly classifying patients as high risk and receiving unnecessary medical interventions. Fifth, the CERT1 score was initially measured at the time of patient admission, and its scores during the follow-up period may be influenced by lipid-lowering and blood glucose lowering medications. Further research is necessary to determine whether changes to the CERT1 score due to these interventions influence its predictive power for patients with ACS undergoing PCI.

## 5. Conclusions

The CERT1 score is currently utilized as a standalone predictor of long-term MACE in individuals with ACS undergoing PCI. However, when combined with the GRACE score, the CERT1 score forms a novel metric that enhances incremental risk-stratification and prognostic value for these patients. This integrative approach not only improves the accuracy of prognostic predictions but also facilitates more informed clinical decision-making, potentially leading to improved outcomes for patients with ACS who have undergone PCI.

## Availability of Data and Materials

The data supporting the findings of this study are not publicly available to protect patient privacy. However, access to the raw data can be requested from the corresponding author as part of reasonable request regulations.
